# Diagnostic and Therapeutic Potential of Exosomal MicroRNAs for Neurodegenerative Diseases

**DOI:** 10.1155/2021/8884642

**Published:** 2021-05-16

**Authors:** Miao He, Hai-nan Zhang, Zhen-chu Tang, Shu-guang Gao

**Affiliations:** ^1^Department of Neurology, The Second Xiangya Hospital, Central South University, Changsha, 410011 Hunan, China; ^2^Department of Orthopaedics, Xiangya Hospital, Central South University, Changsha, 410008 Hunan, China; ^3^National Clinical Research Center of Geriatric Disorders, Xiangya Hospital, Central South University, Changsha, 410008 Hunan, China

## Abstract

Neurodegenerative disorders (NDs) are characterized by a gradual loss of neurons and functions that eventually leads to progressive neurological impairment. In view of the heavy burden on the healthcare system, efficient and reliable biomarkers for early diagnosis and therapeutic treatments to reverse the progression of NDs are in urgent need. There has been an increasing interest in using exosomal miRNAs as biomarkers or targeted therapies for neurological diseases recently. In this review, we overviewed the updated studies on exosomal miRNAs as biomarkers and potential therapeutic approaches in NDs, as well as their association with the pathophysiology of this group of disorders, especially Alzheimer's disease (AD), Parkinson's disease (PD), amyotrophic lateral sclerosis (ALS), and Huntington's disease (HD). The exosomal miRNAs that are commonly dysregulated across different NDs or are commonly used as therapeutic candidates were also identified and summarized. In summary, the feasibility of exosomal miRNAs as biomarkers and potential targeted therapy for NDs has been verified. However, due to the limitations of existing studies and the discrepancies across different studies, high quality laboratory and clinical investigations are still required.

## 1. Introduction

Neurodegenerative disorders (NDs) are a group of diseases that are characterized by a progressive loss of neurons and functions, including Alzheimer's disease (AD), Parkinson's disease (PD), Huntington's disease (HD), and amyotrophic lateral sclerosis (ALS), among others. Due to gradually increased life expectancy, the prevalence of NDs has shown a continuously increasing trend, associated with huge healthcare costs and significant burden on the healthcare system [1]. The current biomarkers of NDs are subjected to deficiencies such as inability to detect changes in the early or preclinical stage of disease [2, 3]. Besides, few or no effective therapeutic treatments are available to reverse or cure this group of diseases to date [4]. Therefore, reliable and easily obtainable biomarkers, as well as effective therapeutic approaches, are urgently needed for NDs.

Extracellular vesicles (EVs), which are nanoscale membrane-bound vesicles secreted from cells, consist of exosomes, microvesicles, and apoptotic bodies based on their intracellular origins [5, 6]. Since conventional extraction methods of vesicles are usually unable to isolate different classes of EVs, some studies used the terms “EV” and “exosome” interchangeably, but in this review, we primarily focused on exosomes. Exosomes refer to vesicles originate from multivesicular bodies with a diameter of less than 100 nm (40-100 nm) [7, 8]. The cargo content of exosomes is primarily composed of proteins, lipids, DNA, mRNAs, and microRNAs (miRNAs) and plays multiple simultaneous roles throughout the human body [9]. In the central nervous system, exosomes can be secreted into the extracellular space by neurons, neuroglial cells, and neural stem cells to exert a neuroprotective or neurotoxic role and take part in both normal neuronal physiological processes and pathogenic processes [10–14].

As one of the most important types of molecules that are contained in exosomes, miRNAs are a class of small, noncoding RNAs, usually about 22 nucleotides [15]. An individual miRNA can repress the translation or regulate the degradation of over 100 mRNAs, and one mRNA can be regulated by multiple miRNAs. Thus, miRNAs are involved in most of the key biological processes including cell signaling, neuronal development, maturation, apoptosis, and neural plasticity [16, 17].

In this review, we provided an updated overview on the diagnostic and therapeutic potential of exosomal microRNAs for NDs, as well as their association with the pathophysiology of the concerned group of disorders. Exosomal miRNAs that are commonly dysregulated across different NDs or are commonly used as therapeutic candidates were also identified and summarized.

## 2. Exosomal miRNA as Biomarkers and Exosomal miRNA-Based Therapy

Exosomes can be purified through different strategies such as ultracentrifugation, density gradient separation, and immunoaffinity capture methods, but such strategies may slightly affect the exosomal contents including miRNAs [18, 19]. miRNAs are enriched in exosomes compared to cell free serum and plasma [20] and are actively packed into exosomes. According to existing studies, miRNAs may be selectively sorted into exosomes through several distinct mechanisms [6], such as (1) the miRNA motif and heterogeneous nuclear ribonucleoproteins- (hnRNPs-) dependent pathway [21], (2) the neural sphingomyelinase 2- (nSMase2-) dependent pathway [22], and (3) the 3′-end of the miRNA sequence-dependent pathway [23]. Encapsulation of miRNAs into EVs can protect those miRNAs from degradation or dilution in the extracellular environment. Therefore, exosomal miRNAs are relatively more stable than miRNAs in serum or plasma [24]. After travelling, these EVs can be efficiently taken up by the targeted cells with specific surface ligands. In this way, exosomes can deliver specific miRNA to the target cells and mediate miRNA exchange between cells, thereby playing a role in cell communication and cell signaling [25]. After being transferred into the recipient cells, miRNAs can then modify the gene expression of recipient cells.

**Figure 1:**
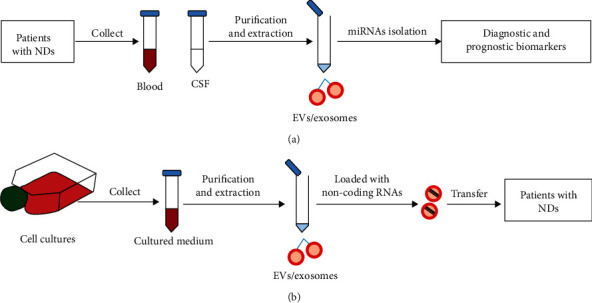
Collection and clinical applications of EVs/exosomes: (a) EVs/exosomes as therapeutic vehicles; (b) EVs/exosomes as diagnostic and prognostic biomarkers.

miRNAs may be used as useful diagnostic and prognostic biomarkers of diseases (Figure 1). The expression levels of exosomal miRNAs vary under different physiological and pathological conditions [2, 6]. Besides, the expression profile of exosomal miRNAs alters in various NDs [26, 27]. Exosomal miRNAs can also be used to discriminate different subtypes of a disease at a high accuracy. For example, the clinical phenotypes of multiple sclerosis (MS) include relapsing-remitting MS (RRMS), secondary progressive MS (SPMS), and primary progressive MS (PPMS) [28]. Ebrahimkhani et al. identified a group of 9 miRNAs that distinguished RRMS from progressive MS. Among the 9 miRNAs, miR-15b-5p, miR-374a-5p, miR-30b-5p, miR-342-3p, and miR-223-3p were expressed in a higher level in RRMS than in S/PPMS, while miR-433-3p, miR-432-5p, miR-23a-3p, and miR-485-3p were the opposite [28]. In general, exosomal miRNAs have been reported to provide an ideal tool as disease-related biomarkers mainly due to the following advantages: (1) they represent the cell of its origin and alter with the disease; (2) they can be measured easily and reliably with a relatively higher specificity and sensitivity; (3) they are detectable in the early stage of diseases; (4) they can be used to distinguish diseases that exhibit similar clinical symptoms, such as PD and AD, and reduce the risk of misdiagnosis; and (5) they can be used to discriminate different subtypes of a disease [6].

Exosomes can be utilized to deliver drugs or genetic elements (Figure 1) [9]. The delivered miRNA may be able to therapeutically alter gene expressions in certain diseases. For example, Yang et al. reported that the rabies viral glycoprotein- (RVG-) exosomes could efficiently deliver miR-124 to the infarct site and promote neurogenesis in ischemic mouse models [29]. The systematically injected exosomal miR-193b-3p could attenuate the neuroinflammatory response in the brains of mice with subarachnoid hemorrhage [30]. Exosomes are deemed promising gene therapy transporters to the brain for the following reasons: (1) exosomes are safe because they do not endogenously replicate [31]; (2) the systematically injected exosomes are able to penetrate the blood brain barrier (BBB) and selectively deliver nucleic acids to the target cells in the brain in a natural way or after modulation [29], and it is feasible to specifically target neural cells by tailoring exosomal membrane proteins [32]; (3) with only biogenic substances, they can travel systemically without stimulating immune responses; and (4) they naturally contain nucleic acids and can protect them from being quickly degraded by ribonuclease in extracellular biological fluids. The miRNA content of exosomes can be modified purposely to enhance their therapeutic effect [29]. Therefore, exosomal miRNAs are attracting increasing attention as potential therapeutic targets for NDs.

### 2.1. Exosomal miRNAs as Biomarkers and the Potential Therapeutic Approach for AD

The clinical manifestations of AD mainly include deterioration of memory, cognitive decline, and changes in personality and behavior. The pathological changes of AD are characterized by the accumulation of amyloid plaques and tau containing-neurofibrillary tangles in the brain of AD patients. The formation of amyloid plaques is caused by overproduction of the amyloid *β* peptide (A*β*). The neurodegenerative changes usually emerge years before the manifestation of clinical symptoms [33].

Dysregulation of miRNAs in the biological body fluid and brain has been found to be associated with the pathological status of AD [34]. In the review conducted by Wu et al., it was concluded that 7 miRNAs (miR-29b, miR-181c, miR-15b, miR-146a, miR-342-3p, miR-191-5p, and let-7d-5p) were consistently downregulated in more than one AD study [35]. miR-107 was consistently downregulated in both AD and mild cognitive impairment (MCI) in two independent cohorts [36, 37]. miR-132 was found to be consistently upregulated in the plasma/serum in MCI patients when compared to normal controls in 3 different studies [38–40]. In addition, some trials have also reached a similar conclusion. However, the results were inconsistent across most of the studies, possibly due to the differences in miRNA stability and concentration in different samples, the diverse analysis procedures used, and the population differences, which hinders the promotion of peripheral miRNAs as biomarkers for AD in clinical practice [2].

miRNAs were significantly enriched and more stable in exosomes when compared to cell-free biofluid samples [20]. Alteration of exosomal miRNA as biomarkers has been reported by several studies in AD patients, suggesting that it could be potentially used to predict AD status at a high accuracy. From the CSF derived exosome samples, a decrease of miR-16-5p in young-onset AD (YOAD, <65 y) but not in late-onset AD (LOAD, >65 y), an increase of miR-125b-5p in both YOAD and LOAD, and a decrease of miR-451a and miR-605-5p in both YOAD and LOAD were detected when compared to controls. These miRNAs target the pathways that are relevant to the molecular processes of AD [41]. Significant differences in miR-9-5p and miR-598 detection rates were found between raw and exosome-enriched AD CSF samples but not in controls, implying different exosome trafficking between AD and control subjects [42]. The expression level of exosomal miR-193b in the CSF and blood samples of AD patients was reduced when compared to healthy controls but not the total miR-193b level in the blood. Meanwhile, negative correlations were observed between exosomal miR-193b and A*β*42 in the CSF of AD patients [43]. A total of 20 plasma exosomal miRNAs showed significant differences in the AD group, among which a panel of 7 miRNAs (miR-185-5p, miR-342-3p, miR-141-3p, miR-342-5p, miR-23b-3p, miR-338-3p, and miR-3613-3p) allows highly accurate prediction of the AD status [44]. Cheng et al. detected 13 serum exosomal miRNAs that were upregulated in AD (Table 1) and 3 miRNAs downregulated in AD (miR-1306-5p, miR-342-3p, and 15b-3p) through sequencing analysis [45]. Many of these miRNAs have been shown to be implicated in the AD pathogenesis based on cell and mouse models [45]. For example, hsa-miR-101 targets the 3′ untranslated region of the amyloid precursor protein (APP) to reduce the APP level and the accumulation of A*β* in human cell lines and hippocampal neurons [46, 47]. The miR-15 family has been observed to regulate tau phosphorylation [48]. miR-424-5p also belongs to the miR-15 family [49]. miR-342-3p is an miRNA that has been proposed as a circulating miRNA biomarker in PD, MS, and Creutzfeldt-Jakob disease [28]. Aforementioned miRNAs were also found to be correlated with the neuroimaging and neuropsychological examination results. However, since the number of MCI participants was small, none of the exosomal miRNAs was verified as a biomarker for predicting disease progression [45]. The level of serum exosomal miR-223, a neuroinflammation-related miRNA, was correlated with the minimental state examination (MMSE) scores and was found to be significantly decreased in AD patients. Besides, the level of miR-223 in AD patients at the first clinic visit was significantly lower than that in AD patients who were currently under medical care, indicating that miR-223 may provide a means to protect nerve cells from apoptosis and can be used to evaluate disease progression [50]. Serum exosomal miR-135a and miR-384 were detected to be upregulated while miR-193b was downregulated in the serum of AD patients when compared to control subjects. Among the 3 miRNAs, the level of serum exosomal miR-384 was significantly higher in AD patients than in VD (vascular dementia) and PDD (Parkinson's disease with dementia) patients, for which miR-384 appeared to be the best choice among the 3 miRNAs for discriminating AD, VD, and PDD. However, the combination of the 3 miRNAs outperformed any particular one for the diagnosis of early AD [51]. Exosomal miR-29c, miR-136-3p, miR-16-2, miR-331-5p, miR-132-5p, and miR-485-5p underwent significant changes in AD CSF when compared to controls [52]. There were 4 miRNAs (miR-23a-3p, miR-126-3p, let-7i-5p, and miR-151a-3p) found to be significantly decreased in AD versus controls. The levels of miR-451a and miR-21-5p were significantly lower in AD samples than in dementia with Lewy bodies (DLB) samples, for which these two miRNAs can help discriminate AD and DLB. The predicted target gene analysis of these miRNAs was associated with protein phosphorylation, proteasomal pathway, and cell death [53]. miR-132-3p and miR-212-3p had significantly reduced levels in neural-derived plasma exosomes in AD and showed good sensitivity and specificity to the diagnosis of AD [54]. Li et al. collected serum EV samples from patients with sporadic AD (*n* = 13), MCI (*n* = 10), and VD (*n* = 10), as well as healthy controls (*n* = 10). The expression levels of 4 proteins and 18 miRNAs in EVs were measured by ELISA or qRT-PCR with the results validated in an independent cohort. The expression levels of miR-1306-5p, miR-342-3p, and miR-15b-3p were significantly decreased in patients with AD when compared to controls, but only miR-1306-5p was downregulated in AD patients (not in VD or MCI patients). A total of 14 miRNAs (Table 1) were significantly upregulated in AD patients when compared to controls, but only the levels of miR-93-5p, miR-424-5p, and miR-3065-5p were upregulated in AD patients (not in VD or MCI patients). Even though the sample size was relatively small, the results indicated that the levels of exosomal miR-1306-5p, miR-93-5p, miR-424-5p, miR-3065-5p, and protein P-S396-tau might be used to differentiate between controls, MCI, or VD patients and AD patients [55]. Cheng et al. analyzed the association between the miRNAs contained in brain-derived EVs (*n* = 8), the miRNAs from matching total brain homogenate, the miRNAs in peripheral EVs (*n* = 23), and controls (*n* = 9). The results suggested a weak correlation between the miRNAs found dysregulated in the brain and periphery blood of AD subjects when compared to controls. However, those miRNAs that were upregulated in both the brain and serum EVs could be used as liquid biopsy for AD diagnosis (Table 1) [56]. By performing high-throughput sequencing, the miRNA cargo of plasma neural-derived small EVs (NDEVs) from 40 AD patients and 40 controls was tested. Further validation showed that the levels of miR-23a-3p, miR-223-3p, and miR-190a-5p in small NDEVs were found to be significantly upregulated whereas the level of miR-100-3p was significantly downregulated in the AD group when compared to healthy controls [57]. The increased expression levels of 13 plasma exosomal miRNAs were found to be associated with decreased Montreal Cognitive Assessment (MoCA) scores in 97 community dwelling older individuals. Among these 13 cognition-related miRNAs, miR-342-3p, miR-125b-5p, and miR-125a-5p were brain selective, while miR-451a-3p showed the highest expression level [58].

Overall, the exosomal miRNAs that were reported to be differentially expressed in the AD group in more than one study include miR-361-5p, miR-30e-5p, miR-93-5p, miR-15a-5p, miR-143-3p, miR-335-5p, miR-106b-5p, miR-101-3p, miR-424-5p, miR-106a-5p, miR-18b-5p, miR-20a-5p, miR-582-5p, miR-1306-5p, miR-342-3p, miR-15b-3p [44, 45, 55], miR-125b-5p [41, 44, 58], miR-139b [43, 51], miR-3065-5p [44, 55, 56], miR-23a-3p [53, 57, 59], and miR-125a-5p [58, 59].

RNA interference has emerged as a new promising therapeutic candidate for the treatment of neurodegenerative disorders [60]. The therapeutic potential of RNA drugs delivered by exosomes has aroused a great interest from an increasing number of researchers. Alvarez-Erviti et al. demonstrated the therapeutic potential of exosome-mediated siRNA delivery by transporting exosomal siRNA that targets *BACE1* (*β*-site amyloid precursor protein cleaving enzyme 1) to the mouse brain. BACE1 is a protease responsible for the N-terminal cleavage of APP and is a well-known therapeutic target in AD. The researchers first modified dendritic cells to express the lysosome-associated membrane protein 2 (LAMP2) and then fused with RVG to target the central nervous system. Furthermore, they loaded the siRNAs of interest to the derived exosomes. After systematic injection, the siRNA was specifically delivered to the neurons, microglia, and oligodendrocytes mediated by RVG targeted exosomes, resulting in the knockdown of *BACE1* and a significant decrease in the total *β*-amyloid 1-42 levels [32]. Sarkar et al. demonstrated the overexpression of miR-34a in specific brain regions of AD patients. The increased level of miR-34a in the temporal cortex was found to be correlated with the severity of AD pathology. The miR-34a-loaded exosomes secreted by miR-34a-overexpressed neurons deliver miR-34a to recipient neurons. In the target neurons, miR-34a can mediate the concurrent repression of its target genes, which may dedicate to the dysfunction in memory circuits [61]. The expression of miR-29 was found to be associated with DNA damage and cell senescence both in normal and pathological aging and would accumulate during the aging process [62]. The miR-29 family is significantly downregulated in AD and is probably involved in the pathogenesis of the disease. Jahangard et al. transfected the rat bone marrow mesenchymal stem cells (MSC) and HEK-293T cells with vectors carrying the precursor sequences of miR-29. They collected the miR-29 enriched exosomes excreted from the cells after confirming the overexpression of miR-29 and the downregulation of their target genes *BACE1* and *BIM* (Bcl-2 interacting mediator of cell death (BCL2-like 11)) in the transfected cells. The miR-29 enriched exosomes were then injected into the cornu ammonis area of A*β*–treated model rats. The spatial learning and memory deficits of model rats were prevented after the treatment, indicating that such engineered miR-29 enriched exosomes may have a therapeutic potential. However, animal models cannot mimic all the features of AD, thereby limiting the implication of the findings [63]. Wei et al. reported that the establishment of an AD cell model was accompanied by increased cell apoptosis and decreased miR-223, while the MSC-derived exosomal miR-223 inhibited the neuronal apoptosis of AD cell model by activating the PTEN-PI3K/Akt pathway [64]. Repetitive mild traumatic brain injury (rmTBI) is deemed an important risk factor for AD. The level of microglial exosomal miR-124-3p from injured brain was altered in different phases after rmTBI. After conducting *in vitro* and *in vivo* experiments, Ge et al. concluded that microglial exosomal miR-124-3p could alleviate neurodegeneration and cognitive deficits after rmTBI by targeting the Rela/ApoE signaling pathway [65]. Even though no overt side effect was reported so far after the execution of exosome-mediated RNA therapy, the long-term impact of the treatment still needs to be further investigated.

### 2.2. Exosomal miRNAs as Biomarkers and the Potential Therapeutic Approach for PD

PD is clinically characterized by resting tremor, rigidity, bradykinesia and postural instability, and various nonmotor symptoms [66]. Exosomes are associated with the spread of *α*-synuclein and inflammatory response and are linked to the progression of PD pathology in the brain [67]. The dysregulated expression of miRNAs is functionally associated with the pathological process of PD, such as *α*-syn overexpression and spread, Lewy body formation, and neuronal apoptosis [68].

So far, a limited number of studies have been published on exosomal miRNAs as biomarkers for PD. Cao et al. selected 24 candidate human miRNAs that had previously been reported as PD biomarkers. They remeasured the levels of those miRNAs in serum exosomes collected from 109 PD patients and 40 controls and concluded that the expression levels of exosomal miR19b, miR24, and miR195 could support the diagnosis of PD. No correlation was found between miRNAs and the demographics of patients (e.g., age, smoking, drinking, and Hoehn-Yahr scale). By using the Targetscan tool (http://www.targetscan.org), they found several gene targets of the 3 miRNAs to be closely related to the pathological process of PD, including Parkin RBR E3 ubiquitin protein ligase (miR-19b), LRRK2/PARK8 (miR-19b), and ATP13A2/PARK9 (miR-24 and miR-195) [69]. Ren et al. found that the decreased level of miR-195 led to the increase of Rho-associated kinase 1 (ROCK1), which further induced the activation of microglia and triggered neuroinflammation in a cell model of PD, suggesting that miR-195 is a potential therapeutic target for PD [70]. Santos et al. identified 2 CSF exosomal miRNA-based biomarker panels for the early diagnosis of PD. One panel comprised 5 microRNAs (Let-7f-5p, miR-27a-3p, miR-125a-5p, miR-151a-3p, and miR-423-5p), with 90% sensitivity, 80% specificity, and 82% area under the curve (AUC) for the differentiation of the cohorts. miR-27a-3p was also reported to be decreased in CSF samples of AD patients [71]. Meanwhile, the investigators also identified a panel (miR-10b-5p, miR-22-3p, miR-151a-3p, and *α*-synuclein) with even higher sensitivity, specificity, and AUC. These panels were shown to be associated with the pathways involved in PD pathogenesis such as ubiquitin mediated proteolysis through computational biology analysis [72]. After testing the samples from 52 PD patients and 48 healthy controls, Yao et al. found that the plasma exosomal miR-331-5p was significantly upregulated in PD patients than in controls, while the exosomal miR-505 was downregulated in PD patients. miR-331-5p was mainly packaged in exosomes rather than in the plasma, while miR-505 was mainly expressed in the plasma [73]. A total of 16 exosomal miRNAs were found to be upregulated, and 11 miRNAs were downregulated significantly in PD CSF when compared to normal controls (Table 1). The results from the microRNA assay were validated by TaqMan Real-Time PCR using independent samples [52]. In a recent study conducted by Nie et al., exosomal miRNAs were extracted from the plasma samples collected from 5 AD patients, 7 PD patients, and 34 controls. It was found that 3 miRNAs (miR-423-5p, miR-369-5p, and miR-23a-3p) were significantly elevated, and 5 miRNAs (miR-204-5p, miR-125a-5p, miR-1468-5p, miR-375, and let-7e-5p) were significantly reduced in AD samples when compared to the control group. Only one miRNA, let-7e-5p, was differentially expressed between PD and control. It was found to be elevated in PD samples and reduced in AD samples, indicating that let-7e-5p can be used as a biomarker to differentiate AD and PD [59]. The expression levels of 23 serum miRNAs were investigated in a cohort of 139 patients including AD, PD, VD, VP (vascular parkinsonism) patients, and healthy controls. The miR-23a showed an increased level in all NDs when compared to controls. The miR-22∗ and miR-29a were dysregulated in both Alzheimer- and Parkinson-like diseases. let-7d, miR-15b, miR-24, miR-142-3p, miR-181c, and miR-222 seemed to be associated with Parkinson-like phenotypes, while miR-34b, miR-125b, and miR-130b exhibited altered expressions only in Alzheimer-like disorders [74]. The investigators also compared the expressions of miRNAs in serum exosomes and in serum without exosomes in small groups of patients (5 patients per group). The results showed that miR-23a and miR-125b were only upregulated in the serum deprived of exosomes, while upregulation of miR-34b was observed only in serum exosomes. On the contrary, miR-29a was upregulated in both serum exosomes and serum without exosomes. The results indicated different distributions of miRNAs inside and outside exosomes [74]. Moreover, the exosomal miR-331-5p was reported to be dysregulated in PD by 2 independent studies [52, 73].

Regarding the potential exosomal miRNA therapy, Kojima et al. introduced a set of EXOsomal transfer into cell (EXOtic) devices that enabled efficient and customizable production of designer exosomes in engineered HEK-293T cells. The introduction of EXOtic devices can largely enhance exosome production, specific miRNA packaging, and delivery into the cytosol of target cells. The therapeutic catalase miRNA from engineered exosome-producing cells was able to attenuate neurotoxicity and neuroinflammation in 6-hydroxydopamine (6-OHDA) or LPS induced mouse models of PD and could open up new RNA delivery-based therapeutic opportunities for the treatment of PD [75]. To assess the ability of exosomes loaded with *α*-Syn siRNA to decrease *α*-synuclein and its aggregates, Cooper et al. developed modified exosomes expressing RVG and loaded them with *α*-Syn siRNA. Then, they peripherally injected those exosomes into normal mice and transgenic mice expressing the human phosphorylation-mimic S129D *α*-Syn. Significant reduction in brain *α*-Syn mRNA and protein levels, as well as in intraneuronal protein aggregates, was detected in both normal and transgenic mice 7 days after treatment with the *α*-Syn siRNA loaded exosomes [76]. The microRNA-124 loaded nanoparticles were reported to be able to promote the subventricular zone (SVZ) neurogenesis, induce the migration of neurons into the lesioned striatum of the 6-OHDA PD mouse model, and improve the motor performance of 6-OHDA mouse after intracerebral administration [77]. The 1-methyl-4-phenylpyridinium (MPP^+^) is a well-known neurotoxin which can be used to induce cell death in PD cell models. Shakespear et al. demonstrated that exosomes derived from normal astrocytes, but not from MPP^+^-stimulated astrocytes, exhibited significant cell-protective effects in MPP^+^-treated SH-SY5Y cells, as well as in primary mesencephalic dopaminergic and hippocampal neuron cultures. They further confirmed that exosomal miR-200a-3p showed the largest reduction among all the miRNAs expressed in MPP^+^-stimulated astrocytes through small-RNA sequencing. The astrocyte-derived exosomal miR-200a-3p was found to attenuate apoptotic cell death in MPP^+^-treated PD cell models and glutamate-treated hippocampal neuron cultures through downregulation of the mitogen-activated protein kinase kinase 4 (MKK4), which is an important upstream kinase in the c-Jun N-terminal kinase cell death pathway [78]. By upregulating OXR1, the downregulation of exosomal miR-137 could inhibit the oxidative stress injury of neurons, promote neuron viability, and inhibit the apoptosis of neurons in both cell models and mouse models of PD [79].

### 2.3. Exosomal miRNAs as Biomarkers and the Potential Therapeutic Approach for ALS

ALS is a progressive, neurodegenerative disorder with poor prognosis, and its core pathological finding is the loss of upper and lower motor neurons [80]. The clinical manifestation of ALS is heterogeneous, mainly including the upper and lower motor neuron features in limbs and chewing, speaking, and swallowing difficulties [81, 82]. The pathophysiological processes underlying ALS reflect an interplay between environmental and hereditary factors [81].

Only a few studies have analyzed the expression of exosomal miRNA in ALS. Downregulation of serum exosomal miR-27a-3p was detected in 10 ALS patients when compared to healthy subjects. miR-27a-3p could be transferred by myoblast exosomes to promote osteoblast mineralization and might be involved in ALS development [83]. Analysis of miRNAs in plasma EVs collected from ALS patients revealed elevated levels of 5 miRNAs (especially miR-532-3p, miR-144-3p, and miR-15a-5p) and reduced levels of 22 miRNAs (especially miR-4454, miR-9-5p, and miR-338-3p) when compared to controls. Some miRNAs that had previously been reported to be relevant to ALS, including miR-9-5p, miR-183-5p, miR-338-3p, and miR-1246, were found to be deregulated. These ALS-relevant miRNAs were speculated to be associated with the processes such as transcriptional regulation and protein ubiquitination [84]. In plasma neuro-derived EVs collected from ALS patients, 13 miRNAs were detected to be significantly upregulated, and 17 miRNAs were significantly downregulated when compared to healthy controls. Gene ontology analysis revealed that the target genes altered by the dysregulated miRNAs were involved in the synaptic vesicle-related pathway. Most of the miRNAs isolated from neuron-derived EVs were found to be overlapped with the miRNAs expressed in the brain tissue. Particularly, 4 miRNAs in plasma neuro-derived EVs (miR-24-3p, miR-1268a, miR-3911, and miR-4646-5p) were found to be regulated in a similar manner to those in formalin-fixed paraffin-embedded motor cortex samples collected from ALS patients. The target genes for the 4 miRNAs partly overlapped in *STX1B*, *RAB3B*, and *UNC13A* genes. More specifically, *UNC13A* has been reported to be associated with an increased risk of sporadic ALS [85].

Bonafede et al. reported for the first time that the exosomes from murine adipose-derived stromal cells were able to protect the NSC-34 cells with ALS mutations from oxidative damage and to increase cell viability [86]. The exosomes derived from adipose-derived stem cells have been demonstrated to be able to modulate SOD-1 aggregation and mitochondrial dysfunction *in vitro* and therefore can be a therapeutic candidate for ALS [87]. Varcianna et al. extracted exosomal miRNAs from human induced astrocytes collected from both ALS patients carrying *C9orf72* mutations (C9ORF72-ALS iAstrocytes) and healthy controls. By setting a threshold with *p* value ≤0.05 and fold change ≥ 1.5, the investigators identified 64 dysregulated miRNAs (51 upregulated and 13 downregulated) in C9ORF72-ALS iAstrocytes. The dysregulated miRNAs had an impact on neurite network maintenance and motor neuron survival *in vitro*, suggesting their involvement in the motor neuron death in ALS. In particular, they detected the downregulation of miR-494-3p, which is an upstream target in regulating axonal maintenance and primarily targets Semaphorin 3A (Sema3A). Restoring the level of miR-494-3p can improve motor neuron survival *in vitro*, which supports miR-494-3p as a potential therapeutic target. However, the difficulty to target specific miRNAs might raise safety concerns for *in vivo* manipulation [88]. Roy et al. concluded that modulation of the inflammatory-associated miR-124 in mutant copper-zinc superoxide dismutase 1 NSC-34 motor neurons and their derived exosomes might be a promising therapeutic strategy for halting motor neuron degeneration in ALS [89].

### 2.4. Exosomal miRNAs as Biomarkers and the Potential Therapeutic Approach for HD

HD is a monogenic, dominantly inherited neurodegenerative disorder that is caused by an abnormal expansion of CAG triplet repeats in the gene *huntingtin* (*HTT*). The main clinical manifestation of HD includes involuntary choreiform movements, cognitive impairment, and neuropsychiatric symptoms [90]. Certain miRNAs were detected as biomarkers in the presymptomatic HD gene expansion carriers when treatments may be the most consequential. For example, miR-520f-3p, miR-135b-3p, miR-4317, miR-3928-5p, miR-8082, and miR-140-5p were significantly increased in CSF in the prodromal HD group [91].

About 50–60% of the dysregulated miRNAs in HD (including striatum of mouse HD models, frontal cortex of monkey HD models, and HD patient brain models) were found in exosomes [92], such as miRNA-128a that targets *HTT* and *HIP1* [93]. Other HD related miRNAs, such as miR-22, miR-214, miR-150, miR-146a, and miR-125b, are all found in exosomes [94, 95]. Therefore, miRNAs in exosomes are linked to HD [92].

miR-124 is one of the key miRNAs that is repressed in HD. The researchers first generated a miR-124-overexpressing HEK-293 cell line, then harvested miR-124-enhanced exosomes from the cells and injected the exosomes into the striatum of R6/2 transgenic HD mice [96]. As a result, the expression of the key target gene, RE1-Silencing Transcription Factor, was found to be reduced. However, the exosome treatment had no effect on Dcx protein levels and the behavioral performance of the treated mice, probably due to the limited therapeutic effect of miR-124 or the insufficient dose of miRNAs packed in the exosomes [96]. The engineered microRNA targeting human huntingtin (miHTT) that is delivered via the adeno-associated serotype 5 (AAV5) virus (AAV5-miHTT) has demonstrated a significant huntingtin lowering effect in vitro and in vivo [97]. Sogorb-González et al. developed EVs containing AAV5-miHTT from induced pluripotent stem cells- (iPSC-) derived neurons. The therapeutic miHTT molecules within EVs can be taken by other HD neuronal cells in a concentration-dependent manner, and the EVs containing AAV5-miHTT may be therefore used for future gene therapy for HD [98].

### 2.5. Exosomal miRNAs Dysregulated in Multiple NDs

Exosomal miRNAs that were found to be dysregulated in more than one NDs include let-7e-5p, miR-151a-3p, miR-423-5p, miR-132-5p, miR-485-5p, miR-29a, miR-29c, miR-136-3p, miR-16-2, miR-331-5p in AD and PD [52, 53, 59, 72, 74], miR-24-3p and miR-338-3p in AD and ALS [44, 84, 85], and miR-27a-3p and miR-127-3p in PD and ALS [52, 72, 83, 84]. For Spinocerebellar Ataxia Type 3 (SCA3), miR-7014 exhibited a reduced level in plasma-derived exosomes but was upregulated in CSF-derived exosomes and may be a potential biomarker of SCA3 [99] (Table 2).

**Figure 2:**
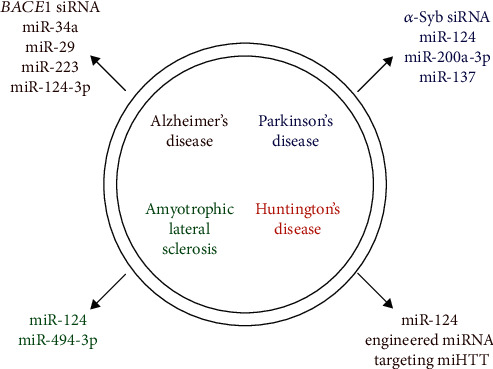
Exosomal miRNAs and siRNAs as potential therapeutic strategy for neurodegenerative disorders. miHTT: human huntingtin.

Exosomal miR-124 has been studied as a candidate therapeutic approach in AD, PD, ALS, and HD models with positive results (Figure 2) [65, 77, 89, 96]. miR-124, belonging to the miRNA family, is abundant in the brain and is critical for neuron differentiation and maintenance [100, 101]. It has also been demonstrated to play a neural protective role in NDs [102, 103]. Since exosomal miR-124 has been reported to be effective in several ND models, it may provide a novel and promising cell-free therapy for patients with different NDs even though more *in vivo* and clinical studies are needed.

## 3. Conclusions

In summary, existing studies have verified the feasibility of large-scale clinical applications of exosomal miRNAs as biomarkers. However, there are still discrepancies across different studies, which are in part due to relatively small sample sizes, as well as the technical variability regarding the purification and extraction of exosomal miRNAs. These limitations may be overcome in the future by enlarging the sample size and improving the purification and extraction methods. Exosomal miRNAs are believed to have the potential to become an effective therapeutic strategy, but existing studies are confined to cell and mouse models only. More comprehensive research including high quality laboratory studies with greater insights into the mechanisms of NDs and large-scale clinical studies are still needed for the discovery and further clinical applications of exosomal miRNAs as biomarkers and potential drugs.

## Figures and Tables

**Table 1:** Exosomal miRNAs as potential biomarkers for neurodegenerative disorders.

Disease	Source	Sample size	Upregulated	Downregulated	ROC curve analysis	References
AD	CSF	P: YOAD: 17 LOAD: 13 C: 12	miR-125b-5p in YOAD and LOAD	miR-16-5p in YOAD; miR-451a and miR-605-5p in both YOAD and LOAD	Y	[41]
	CSF	P: 10 C: 10	miR-9-5p and miR-598 in exosome-enriched AD CSF samples		N	[42]
	CSF	P: 94 C: not indicated		miR-193b	N	[43]
	Plasma	P: 35 C: 35	miR-548at-5p, miR-138-5p, miR-5001-3p, miR-659-5p	miR-23b-3p, miR-24-3p, miR-29b-3p, miR-125b-5p, miR-139-5p, miR-141-3p, miR-150-5p, miR-152-3p, miR-185-5p, miR-338-3p, miR-342-3p, miR-342-5p, miR-3065-5p, miR-3613-3p, miR-3916, miR-4772-3p	N	[44]
	Serum	P: 50 C: 59	miR-361-5p, miR-30e-5p, miR-93-5p, miR-15a-5p, miR-143-3p, miR-335-5p, miR-106b-5p, miR-101-3p, miR-424-5p, miR-106a-5p, miR-18b-5p, miR-20a-5p, and miR-582-5p	miR-1306-5p, miR-342-3p, and 15b-3p	N	[45]
	Serum	P: 32 (AD: 22; VD:10) C: 16		miR-223	Y	[50]
	Serum	P: 208 C:288	miR-135a and miR-384	miR-193b	Y	[51]
	CSF	P: 28 C:27	miR-132-5p and miR-485-5p	miR-29c, miR-136-3p, miR-16-2, miR-331-5p	Y	[52]
	Plasma	P: 10 C:15		miR-23a-3p, miR-126-3p, let-7i-5p, and miR-151a-3p	Y	[53]
	Plasma	P: 32 C:31		miR-132-3p and miR-212-3p	Y	[54]
	Serum	P: 33 (AD:13; MCI: 10; VD: 10) C:10	miR-361-5p, miR-30e-5p, miR-93-5p, miR-15a-5p, miR-143-3p, miR-335-5p, miR-106b-5p, miR-101-3p, miR-424-5p, miR-106a-5p, miR-18b-5p, miR-3065-5p, miR-20a-5p, and miR-582-5p	miR-1306-5p, miR-342-3p, and miR-15b-3p	Y	[55]
	Serum	P: 23 C:20	miR-3157-5p, miR-32-5p, miR-374a-5p, miR-20a-5p, miR-585-5p, miR-941, miR-3065-5p, miR-219a-1-3p et al.		N	[56]
	Plasma	P: 40 C:40	miR-23a-3p, miR-223-3p, and miR-190a-5p	miR-100-3p	N	[57]
	Plasma	P: 5 C: 34	miR-423-5p, miR-369-5p, and miR-23a-3p	miR-204-5p, miR-125a-5p, miR-1468-5p, miR-375, and let-7e-5p	N	[59]
	Serum	P: 54 (AD: 30, VD: 24) C:30	miR-34b, miR-29a		Y	[74]
	Plasma	97 older individuals	miR-342-3p, miR-125b-5p, miR-125a-5p, and miR-451a-3p et al. were associated with decreased MoCA scores		N	[58]

PD	Serum	P: 109 C:40	miR-24, miR-195	miR-19b	Y	[69]
	CSF	P: 40 C:40	let-7f-5p, miR-10b-5p, miR-151a-3p	miR-27a-3p, miR-423-5p, miR-22-3p	Y	[72]
	Plasma	P: 52 C:48	miR-331-5p	miR-505	Y	[73]
	CSF	P: 47 C:27	miR-103a, miR-30b, miR-16-2, miR-26a, miR-331-5p, miR-153, miR-132-5p, miR-485-5p, miR-127-3p, miR-409-3p, miR-433, miR-370, let-7g-3p, miR-873-3p, miR-136-3p, miR-10a-5p	miR-1, miR-22, miR-29, miR-374, miR-119a, miR-126, miR-151, miR-28, miR-301a, miR-19b-3p, miR-29c	Y	[52]
	Plasma	P: 7 C: 34	let-7e-5p		N	[59]
	Serum	P: 55 (PD: 30, VP: 25) C:30	miR-29a		Y	[74]

ALS	Serum	P: 10 C: 20		miR-27a-3p	N	[83]
	Plasma	P: 14 C: 8	miR-532-3p, miR-144-3p, miR-15a-5p, miR-363-3p, miR-183-5p	miR-4454, miR-9-1-5p, miR-9-3-5p, miR-9-2-5p, miR-338-3p, miR-100-5p, miR-7977, miR-1246, miR-664a-5p, miR-7641-1, miR-1290, miR-4286, miR-181b-1-5p, miR-1260b, miR-181b-2-5p, miR-127-3p, let-7c-5p, miR-181a-1-5p, miR-181a-2-5p, miR-199a-2-3p. miR-199b-3p, miR-199a-1-3p	Y	[84]
	Plasma	P: 5 C: 5	miR-4736, miR-4700-5p, miR-1207-5p, miR-4739, miR-4505, miR-24-3p, miR-149-3p, miR-4484, miR-4688, miR-4298, miR-939-5p, miR-371a-5p, miR-3619-3p	miR-1268a, miR-2861, miR-4508, miR-4507, miR-3176, miR-4745-5p, miR-3911, miR-3605-5p, miR-150-3p, miR-3940-3p, miR-4646-5p, miR-4687-5p, miR-4788, miR-4674, miR-1913, miR-634, miR-3177-3p	N	[85]

SCA3	Plasma and CSF	P: 24 C:22		miR-7014 was downregulated in plasma-derived exosomes but upregulated in CSF-derived exosomes	N	[99]

AD: Alzheimer's disease; CSF: cerebrospinal fluid; P: patients; C: controls; YOAD: young-onset AD; LOAD: late-onset AD; ROC: receiver operating characteristic; Y: yes; N: no; VD: vascular dementia; MCI: mild cognitive impairment; PD: Parkinson's disease; VP: vascular parkinsonism; ALS: amyotrophic lateral sclerosis; SCA3: Spinocerebellar Ataxia Type 3.

**Table 2:** Exosomal miRNAs dysregulated in more than one neurodegenerative disorder.

Disease	Exosomal miRNAs	References
AD and PD	let-7e-5p, miR-151a-3p, miR-423-5p, miR-132-5p, miR-485-5p, miR-29a, miR-29c, miR-136-3p, miR-16-2, and miR-331-5p	[52, 53, 59, 72, 74]
AD and ALS	miR-24-3p and miR-338-3p	[44, 84, 85]
PD and ALS	miR-27a-3p and miR-127-3p	[52, 72, 83, 84]

AD: Alzheimer's disease; PD: Parkinson's disease; ALS: amyotrophic lateral sclerosis.
